# *CpLEA5*, the Late Embryogenesis Abundant Protein Gene from *Chimonanthus praecox*, Possesses Low Temperature and Osmotic Resistances in Prokaryote and Eukaryotes

**DOI:** 10.3390/ijms161126006

**Published:** 2015-11-11

**Authors:** Yiling Liu, Lixia Xie, Xilong Liang, Shihong Zhang

**Affiliations:** 1 College of Plant Sciences, Jilin University, Changchun 130062, China; liuyiling21th@126.com (Y.L.); xialixia128@163.com (L.X.); 2 College of Agronomy, Heilongjiang Bayi Agricultural University, Daqing 163319, China; xlliang09@mails.jlu.edu.cn

**Keywords:** *Chimonanthus praecox*, late embryogenesis abundant protein (LEA), low-temperature resistance

## Abstract

Plants synthesize and accumulate a series of stress-resistance proteins to protect normal physiological activities under adverse conditions. *Chimonanthus praecox* which blooms in freezing weather accumulates late embryogenesis abundant proteins (LEAs) in flowers, but *C. praecox* LEAs are little reported. Here, we report a group of five LEA genes of *C. praecox* (*CpLEA5*, KT727031). Prokaryotic-expressed *CpLEA5* was employed in *Escherichia coli* to investigate bioactivities and membrane permeability at low-temperature. In comparison with the vacant strains, *CpLEA5*-containing strains survived in a 20% higher rate; and the degree of cell membrane damage in *CpLEA5*-containing strains was 55% of that of the vacant strains according to a conductivity test, revealing the low-temperature resistance of *CpLEA5* in bacteria. *CpLEA5* was also expressed in *Pichia pastoris.* Interestingly, besides low-temperature resistance, *CpLEA5* conferred high resistance to salt and alkali in *CpLEA5* overexpressing yeast. The *CpLEA5* gene was transferred into *Arabidopsis thaliana* to also demonstrate *CpLEA5* actions in plants. As expected, the transgenic lines were more resistant against low-temperature and drought while compared with the wild type. Taken together, *CpLEA5*-conferred resistances to several conditions in prokaryote and eukaryotes could have great value as a genetic technology to enhance osmotic stress and low-temperature tolerance.

## 1. Introduction

Low-temperature damage is the main factor limiting plant growth and crop production in North and Northeast China. Tolerance to low temperature by overwintering plants accumulates during exposure to temperatures near to but greater than the freezing point [1,2]. This process is called cold acclimation (CA), which develops by activating the expression of particular genes [2]. Gene expression induced by CA has been demonstrated in several plant species, such as *Arabidopsis thaliana* [3] and winter wheat [4]. Tolerance to freezing temperatures has been related to CA-induced gene expression [3,5,6].

Late embryogenesis abundant proteins (LEA) which were first identified and characterized in cotton (*Gossypium hirsutum*) [7,8] are generated in large quantities in seeds through the expression of extremely hydrophilic genes induced by CA [9]. LEA proteins also accumulate substantially in plants under extreme water stress (e.g., low- or high-salt stress and dehydration) [10]. LEA proteins have an extremely hydrophilic structure with a highly free and unordered conformation, and they maintain their solubility after boiling or freezing due to these spatial characteristics [11]. Thus, LEA proteins protect plants under water stress [3,12].

Many new LEA proteins have been identified in various plants, since LEA proteins were first reported in plants in 1981 [8]. LEA proteins are classified into six groups according to the homology of the amino acid sequences and some special motifs [5,13,14].

LEA proteins in the first group (D-19 family) have a highly conserved motif composed of 20 amino acids with multiple copies. These motifs contain many charged amino acids that are strongly hydrophilic [13]. LEA proteins in the second group (D-11 family) have been investigated extensively with respect to water stress. These proteins are also called dehydrins [15,16] and they are rich in glycine and lysine. LEA proteins in the third group are mainly characterized by the ΦΦE/QXΦKE/QKΦXE/D/Q (Φ represents a hydrophobic residue) 11-mer amino acid motif [17].

LEA proteins in the fourth and fifth groups have relatively less conserved domains, and may be related to maintenance of membrane stability.

LEA proteins in the fifth group lack high specificity in their amino acid residues. In 1999 Cuming firstly described the characterized LEA5 with a higher ratio of hydrophobic residues than the other groups. Different from others, the globular domain in LEA5 remained insoluble in water after boiling [18]. Although there a few reports about LEA proteins in the fifth group, the existing data confirm their transcripts in response to abiotic stresses such as salt, drought, and UV light [19]. The fifth group of LEA proteins are located in different organelles. The Rab28 protein in maize is localized in nucleoli of embryo cells [20]. The SAG21 protein of Arabidopsis is located in mitochondria [21].

Less research has been conducted on LEA proteins in the sixth group. The CaLEA6 protein in *Capsicum annuum* displays a protective role under water stress caused by high salt and dehydration [22].

The multiple functions of LEA proteins are closely related to stress resistance in plants. Much progress has been made on research regarding the structure, function, and gene expression of LEA proteins [13,14,15,16,17,18,19,20,21,22,23,24,25].

To date, diverse plant LEAs have been extensively studied, but these proteins mostly originated from no-antifreeze plants. *C. praecox* is found in north mountain forests of China and blooms in winter. *C. praecox* floral organs are able to survive under such freezing weather, and the cold-proof structures involve such substances that enable to keep the viability of these subtle organs and enhance the abiotic stress tolerance in them. Previous studies revealed, the expressions of *C. praecox* LEA proteins, CpLEA1, CpLEA2 and CpLEA3, and confirmed that the expression of LEA proteins in *C. praecox* were higher in mature seeds compared to that in other tissues. During the flowering stage the expression of CpLEAs were abundant in the early stage of the flower development and then decreased until the blooming stage and increased significantly at the senescence stage, and the anti-freezing property relates well with them [23,24]. It can be inferred that the CpLEA proteins play an important role in the abiotic stresses responses.

Hence, the main content of the present study was to identify and characterize a member in the fifth group of LEAs, the *C. praecox LEA5* gene (*CpLEA5*) in different express systems.

## 2. Results

### 2.1. Bioinformatics Analysis of Chimonanthus praecox CpLEA5 Gene and Protein

We obtained the cDNA sequence (288 bp) with a complete open reading frame encoding the CpLEA5 protein containing 95 amino acids, pI = 9.98, *M*w (molecular weight): 10.41299 kDa. CpLEA5 is a group five LEA (LEA5) protein, from a *C. praecox* flower cDNA library constructed with *Escherichia coli* via an expressed sequence tag analysis. Its physicochemical properties were mostly consistent with LEA5 proteins studied previously, indicating that CpLEA5 may share similar biological functions with LEA5 proteins in other species.

**Figure 1:**
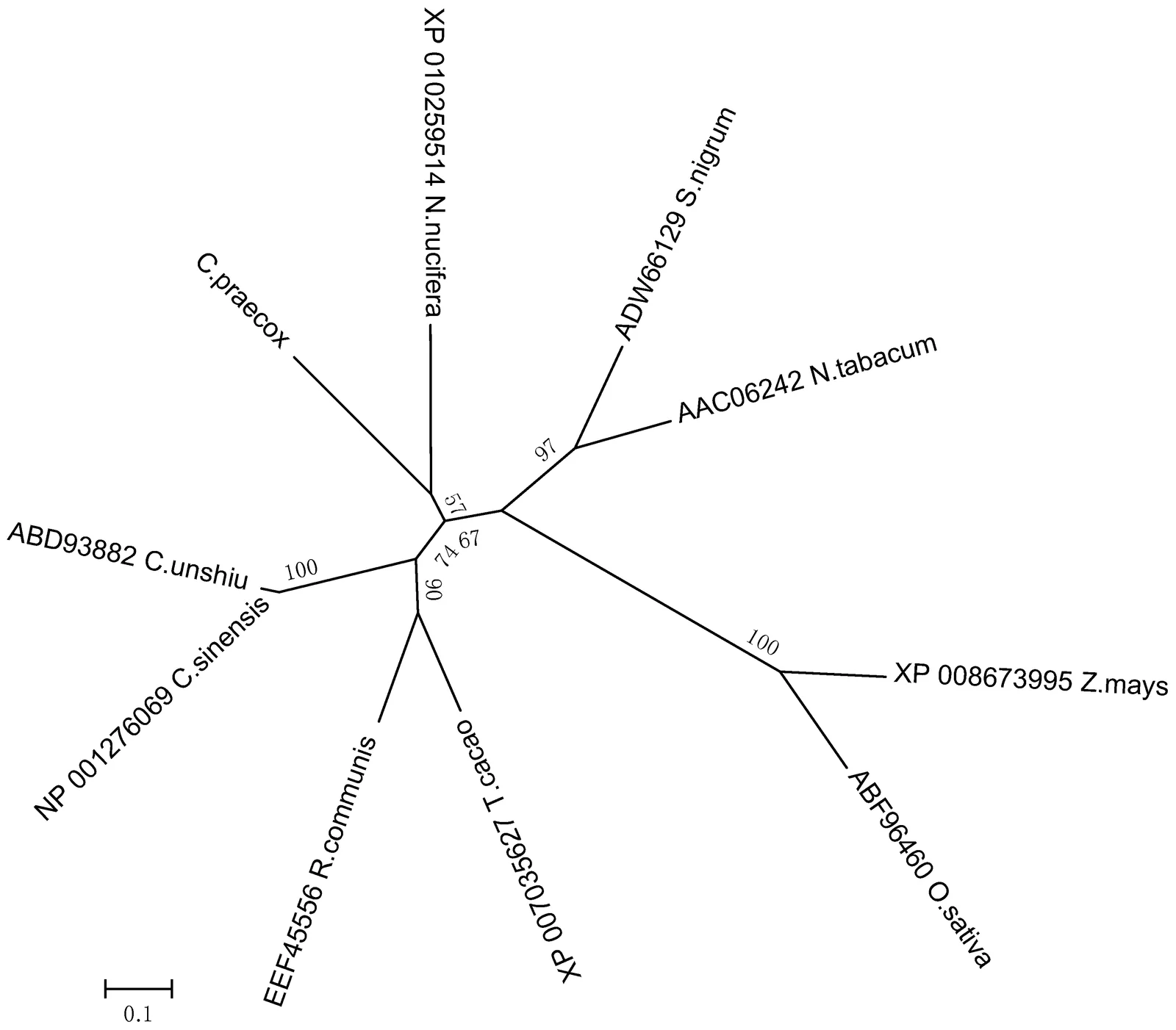
Phylogenetic relationships of CpLEA5 and LEA5 from representative species. A neighbor-jointing tree was constructed using the MEGA6 program. Bootstrap values are shown at the branch points as percentages from 1000 replications. The estimated genetic distance between sequences is proportional to the lengths of the horizontal lines connecting one sequence to another. The protein accession numbers of all LEA proteins are followed by their species names. *C. praecox*, *Chimonanthus praecox*; *T. cacao*, *Theobroma cacao*; *R. communis*, *Ricinus communis*; *C. sinensis*; *Citrus sinensis*; *C. unshiu*, *Citrus unshiu*; *S. nigrum*, *Solanum nigrum*; *N. tabacum*, *Nicotiana tabacum*; *Z. mays*, *Zea mays*; *O. sativa*, *Oryza sativa.*

**Figure 2:**
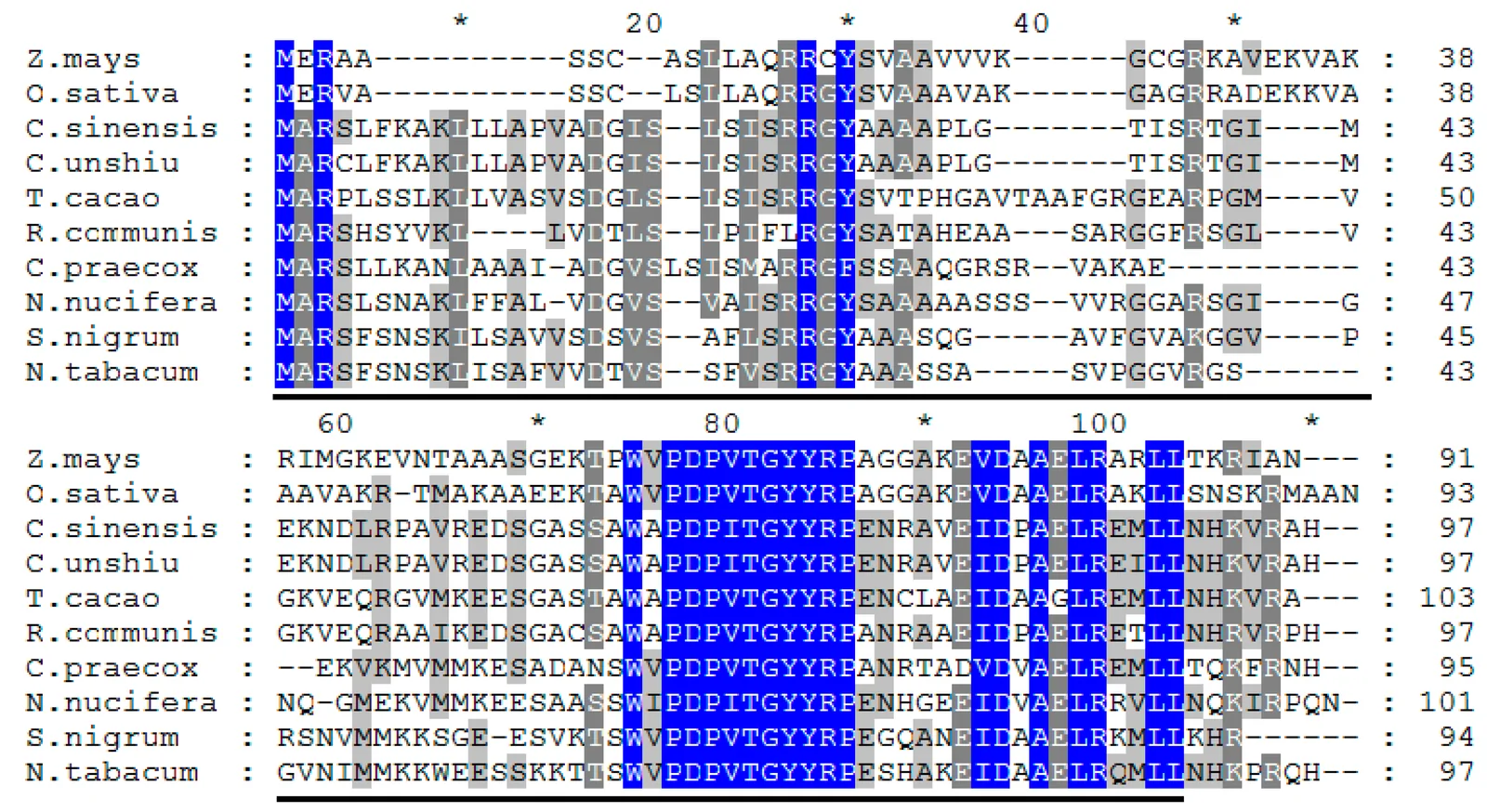
Multiple alignment of *CpLEA5* and *LEA5* from other species in NCBI. Identical residues are shown in blue, highly similar residues are shown in dark grey and similar residues are shown in light grey. The LEA_5 domain is underlined. * Represents the amino acid number increased by twenty from the 10th amino acid.

Analyzing the genetic relationships between *CpLEA5* in different species could provide clues to characterize the functional evolution of *CpLEA5*. Therefore, a phylogenetic tree was generated (neighbor-joining method). It (Figure 1) revealed that the CpLEA5 and the fifth group LEA homologs from *Nelumbo nucifera* were in the same subgroup. Amino acid sequence alignment showed that CpLEA5 shared high similarity (53.26%) with the fifth group LEA protein from *N. nucifera* (Supplementary materials Table S1), indicating a close genetic relationship between these proteins, that may share similar biological functions. In addition, the conserved LEA_3 domain, which is in the LEA5 family, was also recognized in CpLEA5 using the Smart website and Clustal Omega (Figure 2).

### 2.2. Prokaryotic Expression of the CpLEA5 Gene and Anti-Freezing Activity Test of the Expressed Protein

In this section, we investigated whether the *CpLEA5* gene would confer low-temperature tolerance in *E. coli* by comparing the survival rate of the *CpLEA5* gene containing *E. coli* and the empty vector strain after low-temperature treatment.

**Figure 3:**
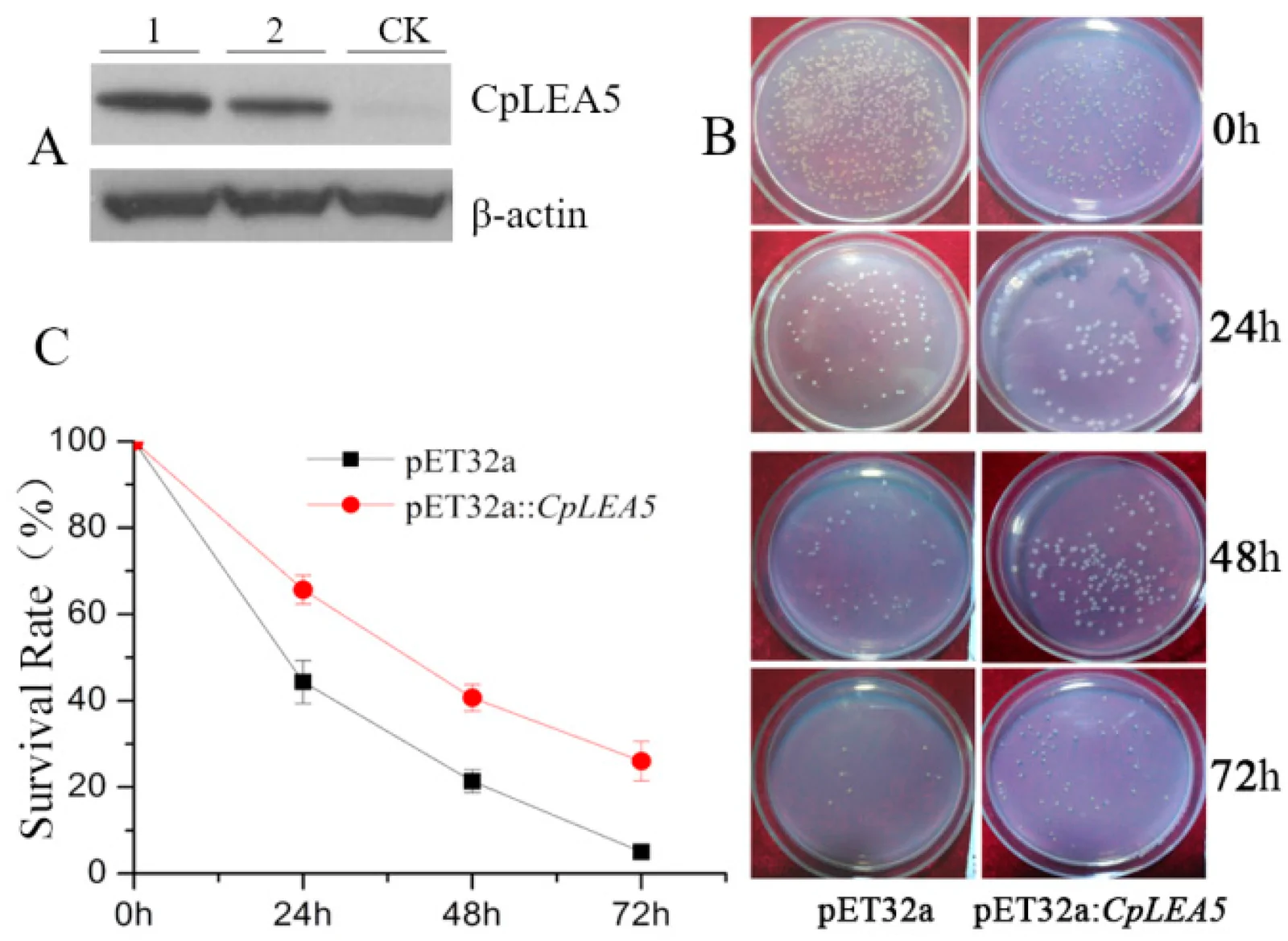
Low temperature resistance assay in *CpLEA5*-overexpressed *E. coli strains.* (**A**) Western-blot analysis of the *CpLEA5*-overexpressed *E. coli* strains. 1 and 2 represent protein numbers extracted at different times; CK represent empty-vector strains; (**B**) Survival comparison between the *CpLEA5*-overexpression strains and the vacant; (**C**) Antifreeze activity Survival Rate curve. Data are means ± standard deviations of three replications. Vacant pET32a strains survival rate as control.

The *CpLEA5* gene fragment was inserted into the pET-32a (*E. coli* prokaryotic expression vector) to construct the pET-32a::*CpLEA5* recombinant plasmid (Supplementary materials Figure S1A), which was transformed into *E. coli* to perform low-temperature tolerance. Western-blot was used to verify the expression of *CpLEA5* protein in *E. coli* (Figure 3A).

Single colonies of a positive clone harboring *CpLEA5* and empty-vector strains were shake cultured in liquid Luria-Bertain (LB) medium. IPTG (isopropyl-β-d-thiogalactoside) was added to induce expression of CpLEA5 protein. Some of the culture medium was removed (positive clone harboring *CpLEA5*, empty-vector strains respectively), and cultured on LB plates at 37 °C, 14 h for no low-temperature treatment. A 1 mL aliquot of the culture medium was removed and placed at −20 °C for 24, 48, and 72 h (positive clone harboring *CpLEA5*, empty-vector strains respectively) for low-temperature treatment. After the treatment, these samples were cultured on LB plates, at 37 °C, 14 h. The bacterial colonies were counted the next day (Figure 3B), and survival rates were calculated as the ratio of the number of colonies after stress to that in the no low-temperature treatment group.

The *CpLEA5* containing strains under a low temperature stress of −20 °C for 24, 48, and 72 h had higher survival rates than the empty-vector strains (*p* = 0.00034), and the percentage of survival increasing were 21.19%, 19.61%, and 20.02%, respectively (Figure 3C).

**Figure 4:**
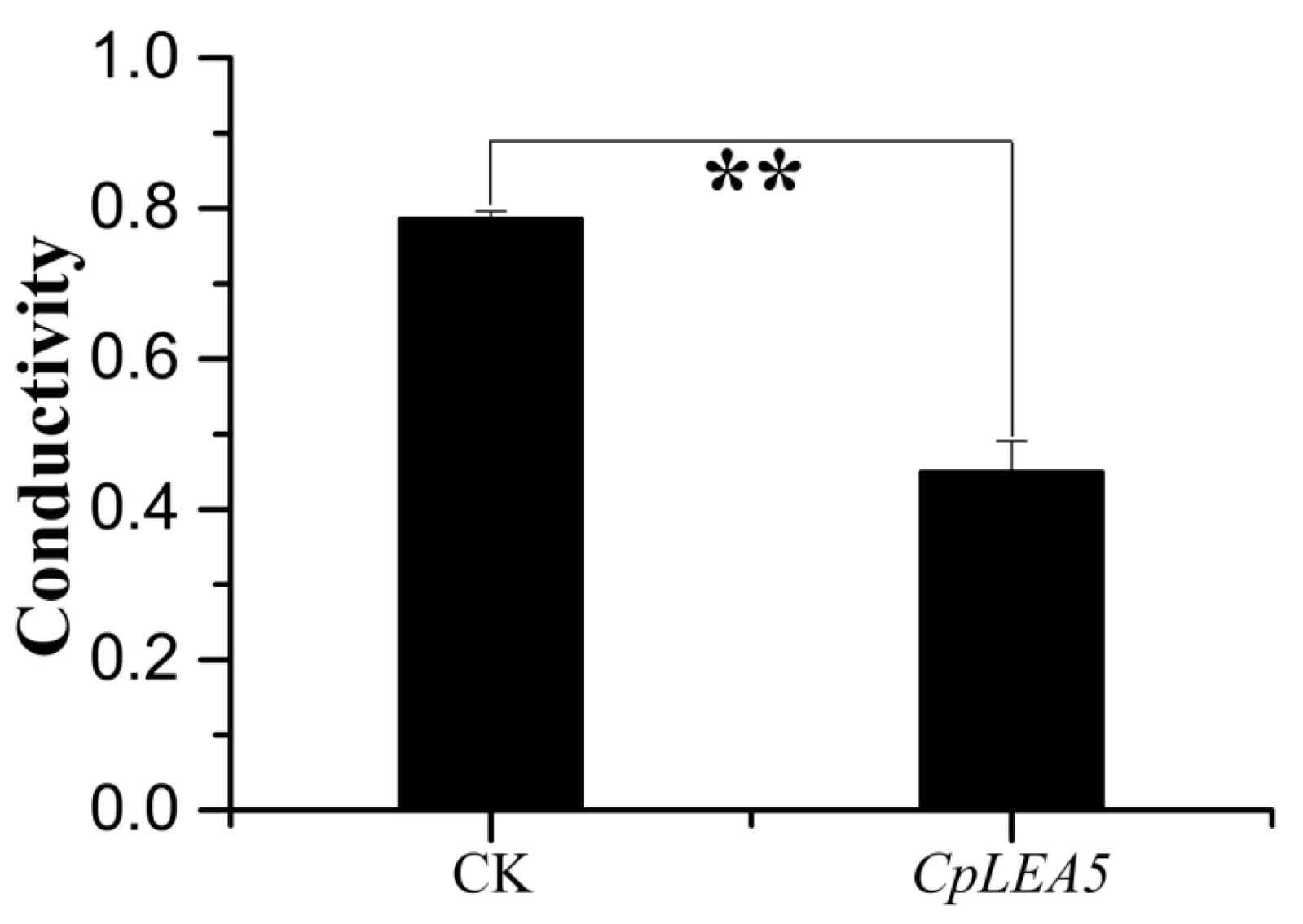
Difference in cell membrane permeability after low temperature treatment at −20 °C for 60 min. Data are means ± standard deviations of three replications. ** Significant difference at *p* < 0.01.

The cytomembrane is important for maintaining the proper microenvironment and normal cell physiology. Cytomembranes are selectively permeable to substances under normal conditions. However, membranes are destroyed and permeability increases when plants are placed under high-temperature, drought, pickling, freezing or other stressors, and intracellular electrolytes diffuse out of the cell. Accordingly, conductivity of a cell extract increases relative to the intensity of cellular stress resistance. Therefore, measuring conductivity is a precise method to rapidly detect cellular stress resistance. We also tested *E. coli* cell permeability under low-temperature stress. The results (Figure 4) indicated that the positive-clone strains harboring *CpLEA5* under low-temperature stress had significantly lower conductivity than that of the empty-vector strains (CK). Conductivity in the positive-clone strains harboring *CpLEA5* was only 55% of that of the empty-vector strains, indicating that membrane integrity of the *CpLEA5* gene-positive clone strains suffered less damage than that of the empty-vector strains. Thus, the *CpLEA5* gene significantly improved low-temperature resistance (*p* < 0.01), demonstrating that the *CpLEA5* gene increased low temperature resistance of the host bacteria.

### 2.3. The CpLEA5 Gene Conferred Stress Resistance in Yeast Expression System

**Figure 5:**
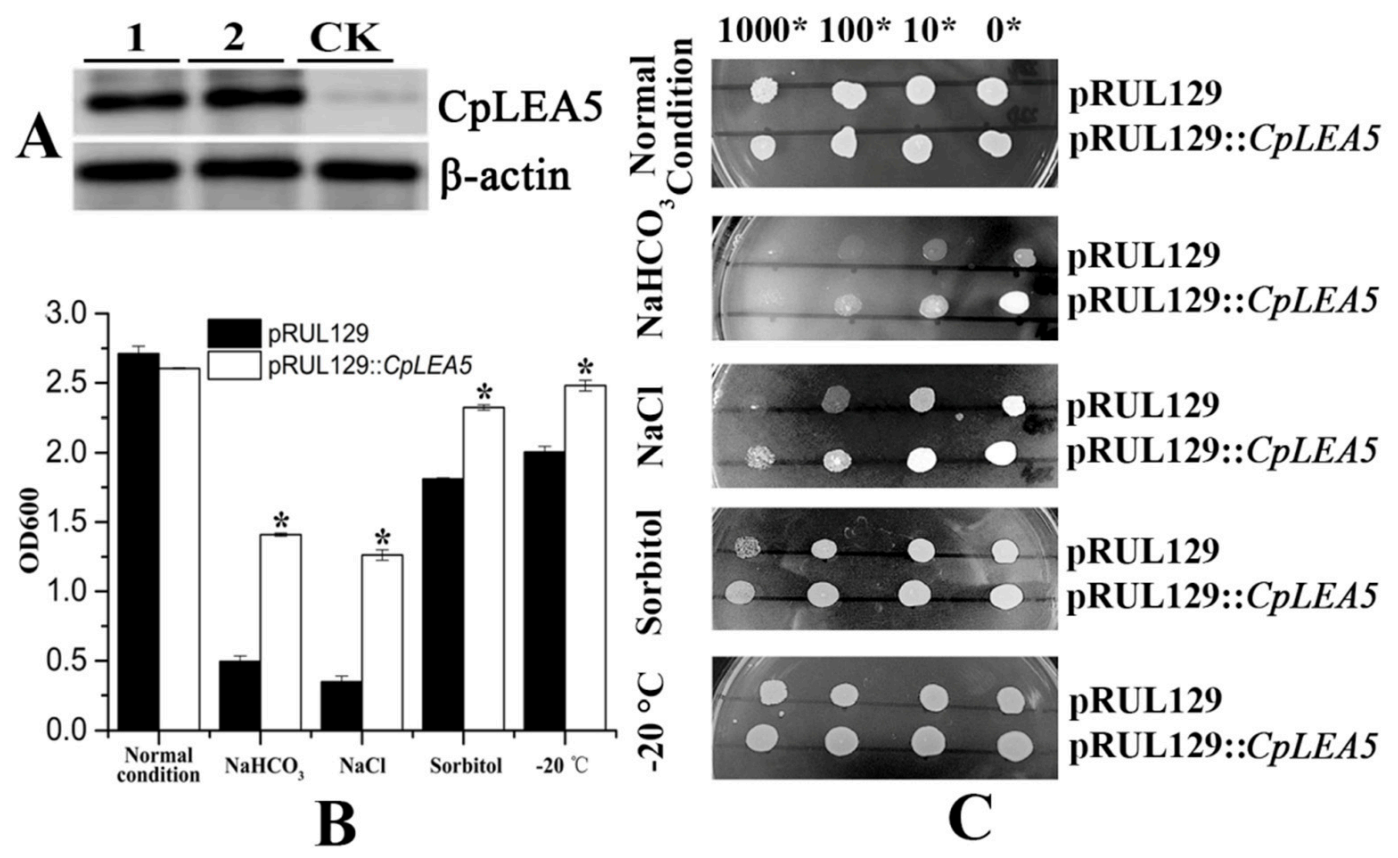
Abio-stress resistance assay in *CpLEA5*-overexpressed yeast. (**A**) Western-blot analysis of the *CpLEA5*-overexpressed yeast; 1 and 2 represent protein numbers extracted at different times; CK represent empty-vector strains; (**B**) OD_600_ value of yeast transformants in response to different abiotic stressors. Data are means ± standard deviations of three replications. * Significant difference at *p* < 0.05; (**C**) Growth of yeast transformants in response to different abiotic stressors. A control group remained untreated without stress.

Yeast is an optimal eukaryotic expression system to rapidly test stress-resistance genes. We rebuilt the pRUL129::*CpLEA5* yeast expression vector (Supplementary materials Figure S1B) and transformed it into the GS115 yeast strain to further verify the stress resistance conferred by the *CpLEA5* gene. The pRUL129 empty vector transformed strain was used in a comparison to determine whether the *CpLEA5* gene is capable of enhancing low-temperature and osmotic resistance in yeast cells. pRUL129::*CpLEA5*-positive strains were identified by Western blot (Figure 5A).

The yeast strains transformed with the pRUL129 empty vector and the pRUL129::*CpLEA5* recombinant expression vector were incubated in solid and liquid SC-U medium (2% galactose) for various stressors subjection (Figure 5B,C).

In normal condition solid and liquid SC-U media, the growth of two kinds of yeast transformants were much the same, mean optical density 600 (OD_600_) value were 2.71 *vs.* 2.60.

Yeast cells expressing the *CpLEA5* gene grew significantly better than those with the empty vector on SC-U medium containing 20% NaCl and medium containing 10% NaHCO_3_. In 10% NaHCO_3_ liquid SC-U medium, mean OD_600_ value of empty-vector strains was 0.49 whereas positive strains harboring *CpLEA5* was 1.4. In 20% NaCl liquid SC-U medium OD_600_ value of empty-vector strains and positive strains harboring *CpLEA5* were 0.34 *vs.* 1.26; the latter is triple the former. The results indicated that the *CpLEA5* gene enhanced the resistance of yeast cells to alkali and salt stressors.

Growth of yeast cells transformed with *CpLEA5* gene on solid SC-U medium containing 2 M sorbitol or after −20 °C low-temperature treatment were slightly better than that of cells transformed with the empty vector, the clone size of yeast cells expressing the *CpLEA5* gene were larger than empty-vector strains. In 2 M liquid SC-U sorbitol media mean OD value of empty-vector strains was 1.81 whereas positive strains harboring *CpLEA5* was 2.32. For after −20 °C low-temperature treatment, mean OD value were 2.0 *vs.* 2.48. This indicates that the *CpLEA5* gene improved drought and low-temperature stress resistance of yeast cells.

### 2.4. Analysis of the Stress Resistance Conferred by the CpLEA5 Gene in Plants

**Figure 6:**
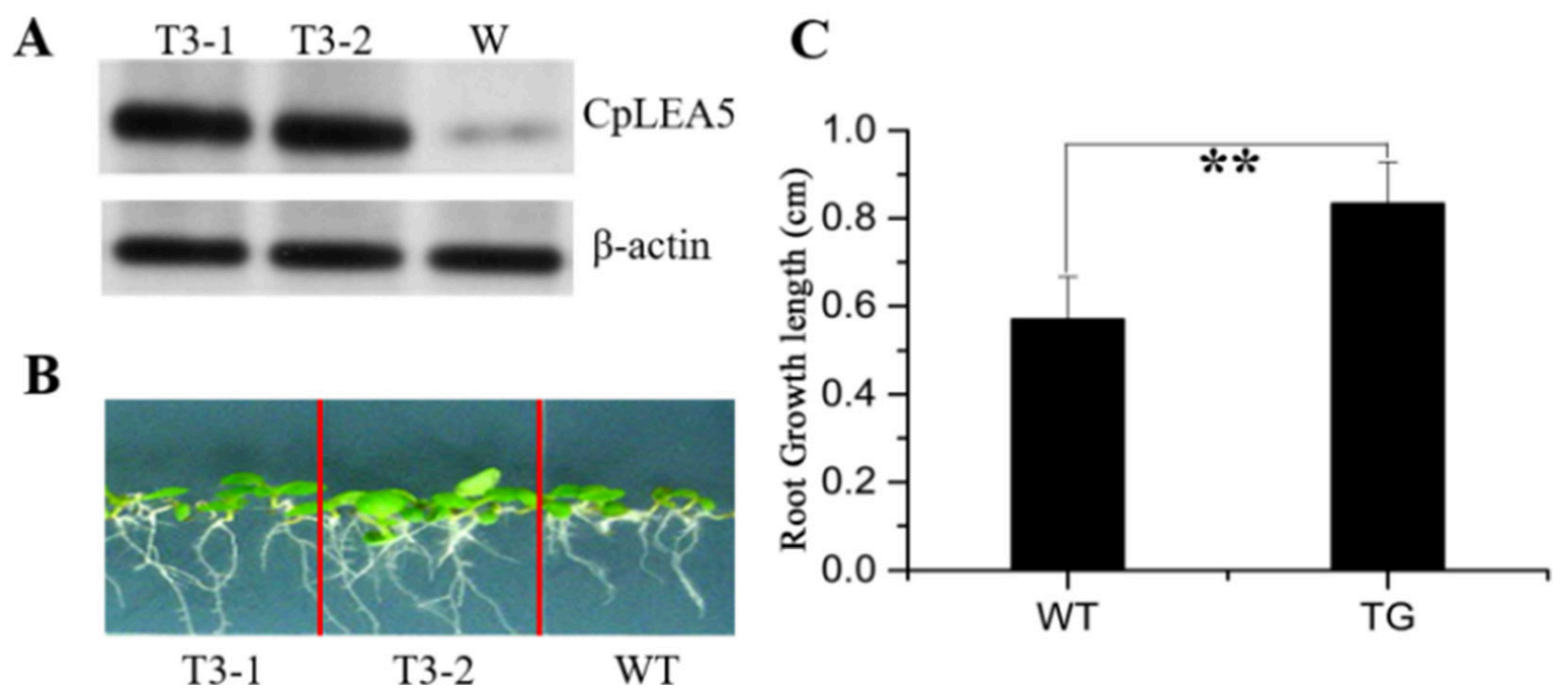
Drought resistance assay in *CpLEA5*-transformed Arabidopsis plants. (**A**) Western-blot analysis of the two independent transgenic lines (T3-1, T3-2) and wild-type line (W); (**B**) Drought stress simulated with mannitol; (**C**) Root growth length after seven days drought stress. Data are means ± standard deviation of three replications. ** Significant difference at *p* < 0.01. WT, wild-type; TG, transfornant.

The pTEV7::*CpLEA5* plant expression vector (Supplementary materials Figure S1C) was constructed to determine whether the *CpLEA5* gene increases abiotic stress tolerance in plants. Using leaf total protein of *Arabidopsis* plants containing the *CpLEA5* gene as the antigen, transgenic *Arabidopsis* plants were selected randomly for treatment under salt-free conditions, and wild-type *Arabidopsis* was used as the negative control. Western blot assay revealed that the immune signal of wild-type *A. thaliana* was significantly weaker than that of *Arabidopsis* with the *CpLEA5* gene, indicating that the transferred *CpLEA5* gene expressed LEA5 protein though an *Arabidopsis* LEA5 homologous to *CpLEA5* (Figure 6A).

Normal growing 7 days-old wild-type *Arabidopsis* and the *CpLEA5* gene-carrying *Arabidopsis*, with mean root lengths of 1.02 and 1.06 cm, respectively, were transplanted into 1/2 Murashige and Skoog (MS) medium containing 300 mM mannitol and cultured for an additional 7 days at 22 °C. Root length was measured to determine growth rate of the plant. The roots of *CpLEA5* gene-carrying *Arabidopsis* were significantly longer after 7 days than those of wild-type *Arabidopsis* (mean length, 1.897 *vs.* 1.593 cm) (Figure 6B). Mean growth length of wild-type *Arabidopsis* roots after 7 days was 0.57 cm whereas the *CpLEA5* gene-carrying *Arabidopsis* was 0.83 cm (Figure 6C). The growth rate of the former was 56%, of the latter was 78%.

**Figure 7:**
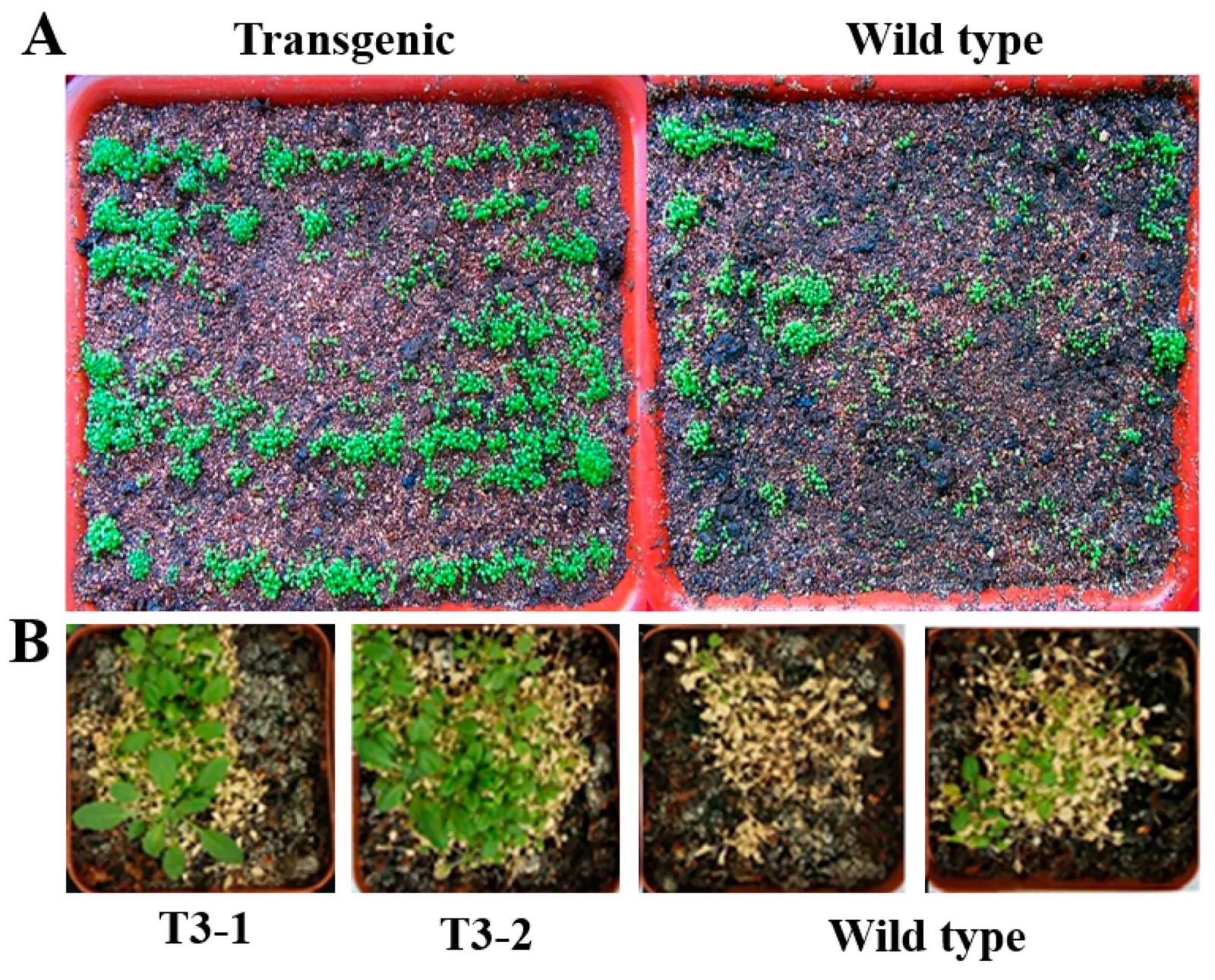
Low temperature resistance in wild type and transgenic plants. (**A**) The seven days old *CpLEA5* gene-carrying *Arabidopsis* line T3-2 and wild type cultured at 4 °C environment for three weeks; (**B**) The seven days old *CpLEA5* gene-carrying *Arabidopsis* line T3-2, T3-2 and wild type cultured at 4 °C environment for six weeks.

Seven-days old wild-type and *CpLEA5* gene-carrying *Arabidopsis* plants grown at 22 °C were transferred to a 4 °C environment for low-temperature stress resistance. After the 3 week culture at 4 °C, growth of the *CpLEA5* gene-carrying *Arabidopsis* was generally better than that of wild-type *Arabidopsis* (Figure 7A)*.* Furthermore, after the 6 week culture at 4 °C, the *CpLEA5* gene contained *Arabidopsis* plants continued to grow, whereas the wild type plants ceased to grow or died (Figure 7B), confirming that the transgenic seedlings overexpressing the *CpLEA5* gene were more drought and cold-resistant than the wild-type seedlings.

## 3. Discussion

Abiotic stress (osmotic, low temperature, *etc.*) is a main factor affecting crop production. In order to ensure their own survival and reproduction, plants developed a series of countermeasures, such as gene expression regulation, to cope with the adverse environment by a range of physiological adaptations. Late embryogenesis abundant (LEA) gene family is closely associated with stress tolerance. The largest class of LEA proteins and homologs are synthesized during the late embryogenesis phase, just prior to seed desiccation in plants [11]. Thus, it is not difficult to imagine that most of LEAs in plants are associated with water stress resistance. To date, many LEA genes have been identified in different plant species, and most of them have been demonstrated to be associated with tolerance against water deficiency, osmotic and freezing stresses [25,26,27,28,29,30]. Moreover, several studies have reported that transferring a LEA gene into plants results in increased osmotic resistance of the transgenic plants [31,32] also. A previous study showed that *WCI16* gene, a LEA protein cloned from wheat could exert protection of both proteins and DNA during environmental stresses after introduction into *Arabidopsis thaliana* [33]. The group four LEA genes, which were isolated from *Boeahygrometrica*, were transferred successfully into tobacco, and transgenic plants possessed enhanced drought resistance [34]. Like *WCI16*, the transgenic tobacco also showed stronger resistance to drought and salt stressors in comparison with the wild type strain.

When exposed to low temperatures, many plants, especially some crops such as rice, maize and soybean, which are native to warm habitat, exhibit symptoms of injury [35]. However, *C. praecox* is very adaptable to different environments. This species suffers relatively few diseases and pathogenic pests but is prone to drought and low temperature stress. Therefore, we speculate that LEA proteins in *C. praecox* is vastly capable of resistance to abiotic stresses. We identified and characterized a *C. praecox LEA5* gene. The *CpLEA5* gene was cloned from a *C. praecox* flower cDNA library using the expressed sequence tags of *C. praecox* flowers. Both prokaryotic and eukaryotic *CpLEA5* gene expression systems were constructed. After expression, better low-temperature stress resistance than that in the wild type was observed. It was demonstrated that the *CpLEA5* gene *can* enhance freezing resistance in both prokaryotic and eukaryotic expression hosts, and we can conjecture that the increase in low-temperature stress resistance in prokaryote and eukaryotes are quite conserved. Moreover, according to our results of measurement of *E. coli* membrane permeability after low-temperature stress, we also speculate that, the CpLEA5 protein may be related to the maintenance of membrane stability. In addition to the low-temperature resistance, both yeast and plant transformants were more resistant to osmotic stress, confirming the multiple resistant abilities of the *CpLEA5* gene.

In contrast to that described above, no change in drought resistance of tobacco was reported after introducing two dehydrin genes and one group three LEA protein gene from the resurrection plant, *Craterostigma plantagineum*single [36]. It was suggested that not all LEA proteins are required for stress resistance in plants; or these LEA proteins require certain factors to activate their resistance functions under stress [37,38].

Krogh *et al*. demonstrated that LEA proteins are not transmembrane proteins but are located in subcellular fractions, such as mitochondria, chloroplasts, nucleus, and cytoplasm [39]. The subcellular locations may vary with the LEAs from different species; for example, group three LEA proteins of nematodes are uniformly distributed in the cytoplasm [40]. Group five LEA proteins have no typical N-terminal signal peptide. *CpLEA5*, as a multiple resistant gene, its subcellular location should be strengthened.

In summary, unlike previous studies in which the LEA5 genes are from plants growing in non-extreme environments (*Arabidopsis thaliana*, maize or cotton), the *CpLEA5* gene in this study is from *C. praecox* which can bloom under a freezing environment. Thus, we can reasonably predict that the *CpLEA5* gene may be used to prolong the growth cycle of the crops in cold regions.

## 4. Experimental Section

### 4.1. Materials and Organism Growth Conditions

The *Escherichia coli* strain DH5α (Invitrogen, Carlsbad, CA, USA), used for *Prokaryotic* expression system was cultured in LB medium. The yeast strain GS115 (*Pichia pastoris*) (Invitrogen, Carlsbad, CA, USA) used in the yeast expression system was grown in SC-U medium containing 2% galactose. *Agrobacteriumtumefaciens* (LBA4404) and *Arabidopsis* (Columbia-0) came from our laboratory (Changchun, China). Both wild type *Arabidopsis* (Columbia-0) and transgenic *Arabidopsis* grown in green house set at 22 °C, with 16 h light, 8 h dark, humidity 75%.

The PCR amplication system and restriction enzyme used in molecular cloning, including BamHI, SacI, HindIII and XhoI were purchased from Takara (Dalian, China). Primers were synthesized and DNA sequencing is performed by Sangon Biotech (Shanghai, China).

*Escherichia coli* strain apoplastic protein, *Pichia pastoris* apoplastic protein, *Arabidopsis* protein were extracted by CelLytic™ B Plus Kit; CelLytic™ Y Plus Kit; Plant Total Protein Extraction Kit separately. The kits above and BCA Protein Assay Reagents were all purchase from Sigma, St. Louis, MO, USA.

### 4.2. Isolation of the CpLEA5 Gene and the Bioinformatics Analysis

In this study, a *Escherichia coli* expression library was constructed by our group, and contained cDNA prepared from treated *C. praecox*. We obtained the *CpLEA5* gene (KT727031) from a *C. praecox* expressed sequence tag analysis. The *CpLEA5* gene fragment was identified using polymerase chain reaction (PCR) amplification, forward primer 5′-CGGGATCCATGGCTCGCTCTCTGTTG-3′, (*Bam*HI sit underlined); reverse primer 5′-CGAGCTCGTGGTTACGGAATTTCTGGG-3′, (*Sac*Isit underlined). The PCR was performed by 35 cycles of PCR (94 °C, 50 s; 54 °C, 30 s; 72 °C, 60 s) Purified PCR productions were loaded into vector pMD-18T vector and identified by sequencing. The pET-32a::*CpLEA5* recombined vector used for prokaryotic expression system was constructed in the same way.

We used online ProtParam tool (http://www.expasy.org/tools/protparam.html) [41] for CpLEA5 molecular weight and isoelectric point prediction. MEGA6.0 [42] was used to generate the phylogenetic tree. Multiple sequence alignment was performed using Clustal Omega (http://www.ebi.ac.uk/Tools/msa/clustalo/) [43] and GeneDoc3.2 [44] alignment programs. Important functional domains were identified using the smart website (http://smart.embl-heidelberg.de/) [45].

### 4.3. Western Blotting Analysis

Western bloting was performed by transferring protein form sodium dodecyl sulfate-polyacrylamide gel electrophoresis (SDS-PAGE) to a polyvinylidenedifluoride (PVDF) membrane (BioRad, CA, USA). Protein concentration was determined with BCA Assay Reagents. Equal quantities protein were separated by SDS-PAGE gel electrophoresis, and transferred to the PVDF membranes. The PVDF membrane was blocked with defatted milk, then incubated with primary antibody (rabbit, Synthesized from Sangon, Shanghai, China) 4 °C overnight, a 2 h secondary antibody (goat anti-rabbit) 1:1000 at room temperature followed by. The protein bands were detected with Super Signal Ultra Chemiluminescent Substrate (Pierce, Rockford, IL, USA) on X-ray films (Kodak, Tokyo, Japan).

### 4.4. Prokaryotic Expression of the CpLEA5 Gene and Measurement of E. coli Membrane Permeability after Low-Temperature Stress

Single colonies of a positive clone harboring *CpLEA5* gene and empty-vector strains were shake cultured at 200 rpm in 5 mL LB liquid medium with containing 100 μg/mL ampicillin (Amp) at 37 °C, until optical density at OD_600_ (600 nm) reached 0.5. Then, IPTG (isopropyl-β-d-thiogalactoside) was added to a final concentration of 1 mM, and the culture was continued for 3 h. Some of the culture medium was removed, diluted 1:10,000, coated on LB plates with Amp (100 μg/mL), and cultured overnight at 37 °C, for 14 h 37 °C. A 1 mL aliquot of the culture medium was removed and placed at −20 °C for 24, 48, and 72 h. After the treatment, the samples were diluted 1:10,000, coated on LB plates with Amp (containing 100 μg/mL), and cultured at 37 °C, for 14 h. The bacterial colonies were counted the next day, and survival rates were calculated as the ratio of the number of colonies after stress to that in the control group. Survival rate after 24 h low-temperature treatment = colony number after stress/colony number in the group without stress

Membrane permeability was measured using the conductivity method. The positive-clone and empty-vector strains were suspended in deionized water at a ratio of 1:109 du/mL after expression was induced. They were treated at −20 °C for 60 min, and 10 mL of the bacterial suspension was centrifuged 15 min, 4000× *g*. The supernatant was used to measure conductivity A. Two strains (pRUL129::*CpLEA5*-positive strains and yeast strains transformed with the pRUL129 empty vector) were suspended in 10 mL deionized water, boiled at 100 °C, 15 min, then centrifuged. The supernatant was used to measure conductivity B. And the final conductivity is calculated by the following formula,

(1) $$C=\frac{C_{A}}{C_{A}+C_{B}}\times 100\%$$

where, *C* is the final conductivity, *C_A_* is conductivity A, *C_B_* is conductivity B.

### 4.5. Yeast Transformants and Stress Treatments

The *CpLEA* gene was amplified by forward primer 5′-CGGGATCCATGGCTCGCTCTCTGTTG-3′, (*BamH*I sit underlined); reverse primer5′-CAAGCTTTGGTTACGGAATTTCTGGG-3′, (*Hind*III sit underlined). The *CpLEA* gene fragment was inserted in the yeast expression vector pRUL129. The recombined vector pRUL129::*CpLEA5*was introduced into yeast strains.

The osmotic stress treatments were conducted in solid and liquid Simmons Citrate-Ura (SC-U) medium. A control group remained untreated without stress. Single colonies of a positive clone harboring *CpLEA5* gene and empty-vector strains were shake cultured at 200 rpm 30 °C 24 h in 5 mL liquid SC-U medium containing 2% glucose. Then 100 μL aliquots of culture medium were removed in 10 mLs liquid SC-U medium containing 2% galactose and shake cultured at 200 rpm 30 °C 24 h to induce expression CpLEA5 protein. The yeast (containing the pRUL129 empty or pRUL129::*CpLEA5* recombinant vectors) liquid was diluted 1:10, 100, 1000, and 10,000, respectively, at an OD_600_ value of 1.0. Then, 4 μL of the original yeast liquid and the diluted liquid were dropped in order on solid SC-U medium (containing 2% galactose). After a 48 h culture at 30 °C, growth of the two kinds of yeast cells was observed and recorded. On the other side, A 1 mL aliquot yeast (containing the pRUL129 empty or pRUL129::*CpLEA5* recombinant vectors) liquid at an OD_600_ value of 1.0 was removed in 10 mL liquid SC-U medium (containing 2% galactose). After a 24 h treatment at 200 rpm, 30 °C, OD_600_ value of the two kinds of yeast cells was measured.

For the low-temperature treatment part, a 1 mL aliquot yeast culture liquid (at an OD_600_ value of 1.0) was treated at −20 °C for 24 h first, then removed in to normal condition solid and liquid SC-U medium (containing 2% galactose) cultured in the same way as the osmotic stress treatments part.

### 4.6. Plant Transformation and Low Temperature Resistance Analysis

In this part, the *CpLEA5* gene was amplified via PCR using forward primers 5′-CCCAAGCTTTGGCTCGCTCTCTGTTG-3′, (*Hind*III site underlined); reverse 5′-CCG*CTCGAG*TGGTTACGGAATTTCTGGG-3′, (*Xho*I site underlined). The PCR product were digested then cloned into plant expression vector pTEV7 directionally. The recombinant plasmid pTEV7::*CpLEA5* was introduced into *Agrobacteriumtumefaciens* (LBA4404). The transformation into *Arabidopsis* was carry out by using floral dip method [46,47]. *CpLEA5* transgenic-seedlings were selected by MS medium containing kanamycin (40 mg/L). The T3 generation of *CpLEA5* transgenic *Arabidopsis*, and wild-type control lines (w) were sown and grown at 22 °C, with 16 h light, 8 h dark, 75% relative humidity. For drought resistant test, 7 days-old plants were washed by sterile water and transplanted into 1/2 Murashige and Skoog (MS) medium containing 300 mM mannitol and cultured for an additional 7 days at 22 °C; for low-temperature resistant tests, 7 days-old plants grown in soil under normal growth conditions were transferred to a 4 °C environment for 3–6 weeks in light, and watered by fresh Hoagland nutrient solution twice per week.

## 5. Conclusions

In the present study, expression of the *CpLEA5* gene was identified and characterized for the first time. *CpLEA5* possesses at least three kinds of resistances (low-temperature resistance, osmotic resistance and drought resistance), and functions in prokaryotic and eukaryotic organisms, which will provide not only genetic candidates for resistant improvement in engineering but also a typical LEA for further study. Detailed studies on the mechanism of action of *CpLEA5* will help us to better control and use the gene as a potential frost-resistant gene for crops.

## Supplementary Materials

Supplementary materials can be found at https://www.mdpi.com/1422-0067/16/11/26006/s1.
