# Correction: Liu et al. *CpLEA5*, the Late Embryogenesis Abundant Protein Gene from *Chimonanthus praecox*, Possesses Low Temperature and Osmotic Resistances in Prokaryote and Eukaryotes. *Int. J. Mol. Sci.* 2015, *16*, 26978–26990

**DOI:** 10.3390/ijms27167472

**Published:** 2026-08-21

**Authors:** Yiling Liu, Lixia Xie, Xilong Liang, Shihong Zhang

**Affiliations:** 1 College of Plant Sciences, Jilin University, Changchun 130062, China; 2 College of Agronomy, Heilongjiang Bayi Agricultural University, Daqing 163319, China

## Error in Figure

In the original publication [1], errors were present in Figures 5A and 6A of the published version. These errors arose from the inadvertent use of incorrect images during figure assembly. The corrected Figure 5A and Figure 6A appear below. The authors state that the scientific conclusions are unaffected. This correction was approved by the Academic Editor. The original publication has also been updated.

## Figures and Tables

**Figure 5:**
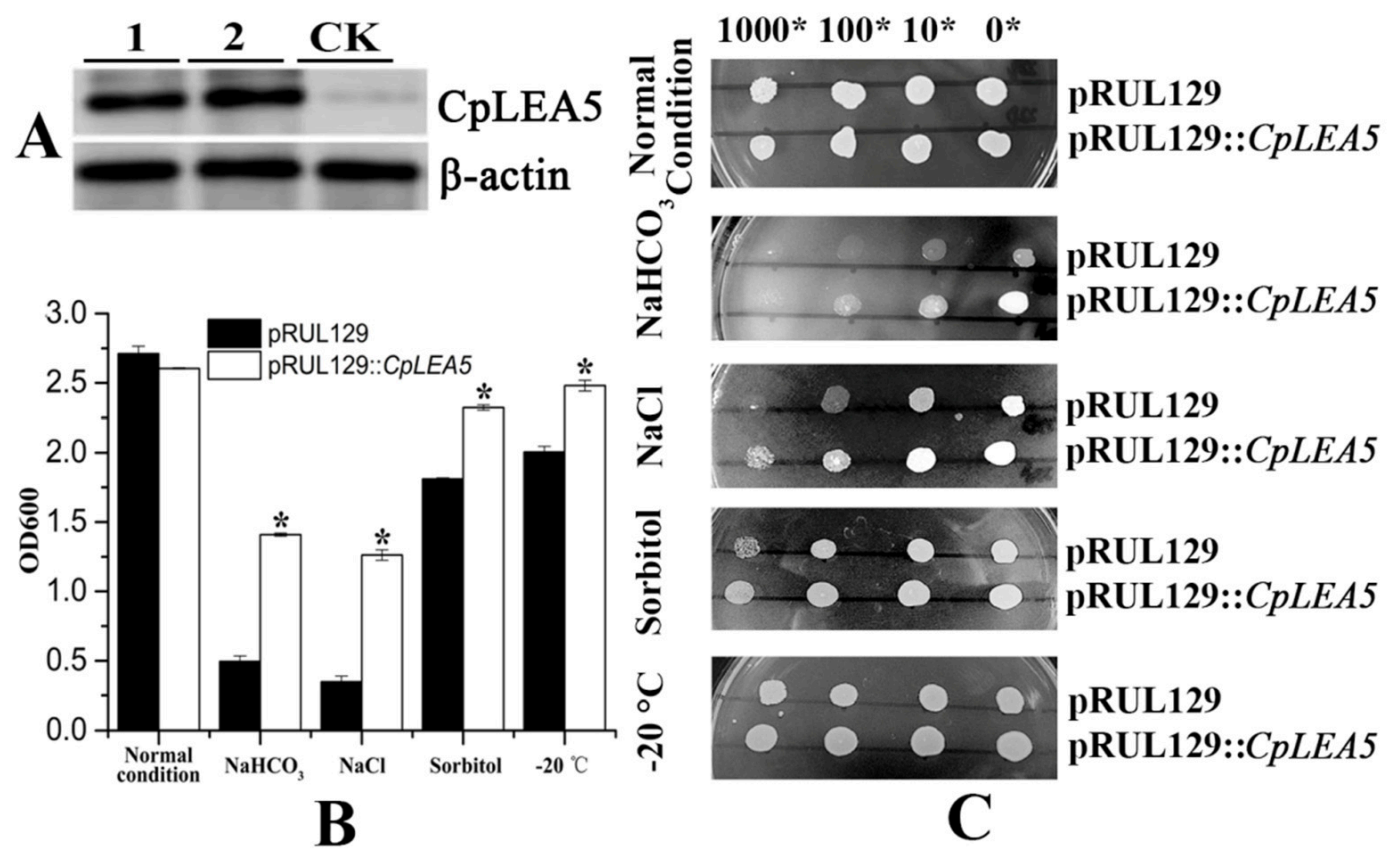
Abio-stress resistance assay in *CpLEA5*-overexpressed yeast. (**A**) Western-blot analysis of the *CpLEA5*-overexpressed yeast; 1 and 2 represent protein numbers extracted at different times; CK represent empty-vector strains; (**B**) OD_600_ value of yeast transformants in response to different abiotic stressors. Data are means ± standard deviations of three replications. * Significant difference at *p* < 0.05; (**C**) Growth of yeast transformants in response to different abiotic stressors. A control group remained untreated without stress.

**Figure 6:**
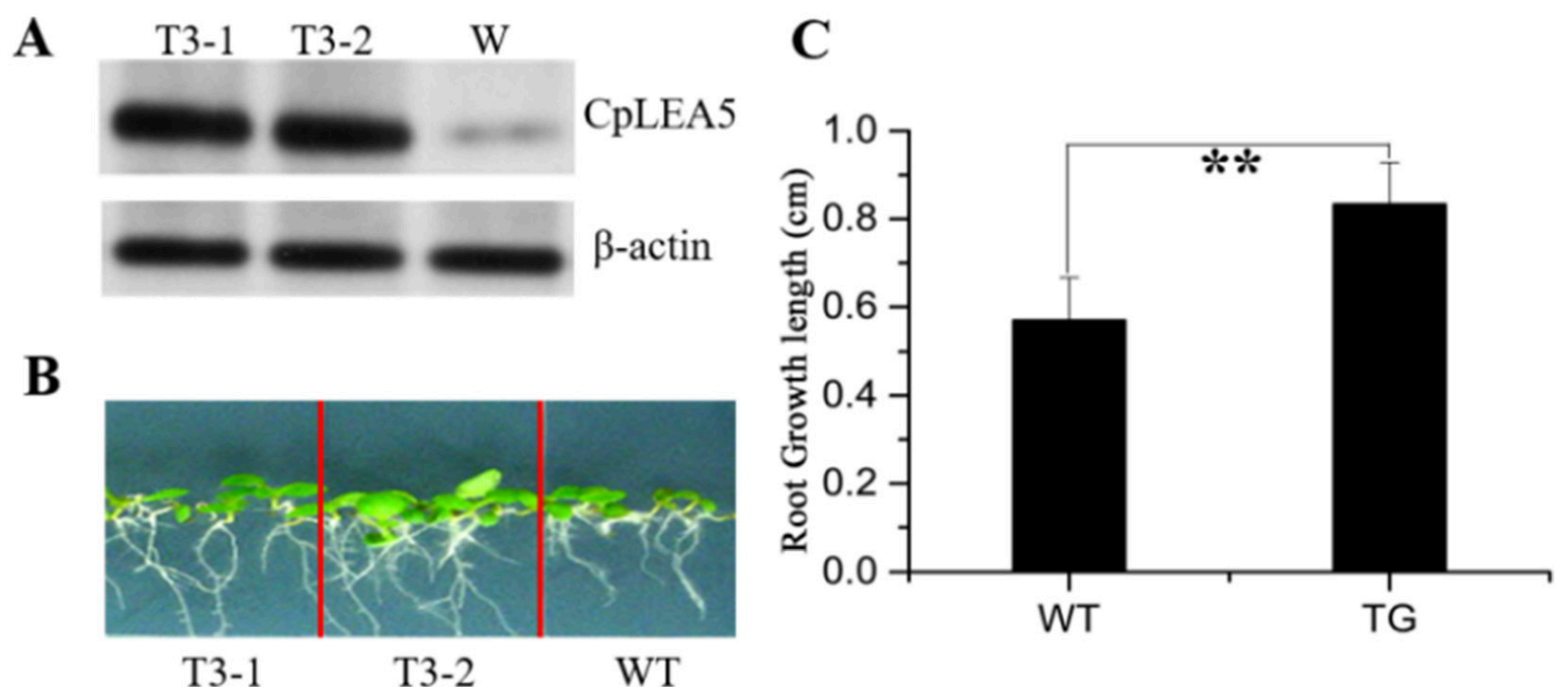
Drought resistance assay in *CpLEA5*-transformed Arabidopsis plants. (**A**) Western-blot analysis of the two independent transgenic lines (T3-1, T3-2) and wild-type line (W); (**B**) Drought stress simulated with mannitol; (**C**) Root growth length after seven days drought stress. Data are means ± standard deviation of three replications. ** Significant difference at *p* < 0.01. WT, wild-type; TG, transfornant.

## References

[1] Liu Y., Xie L., Liang X., Zhang S. (2015). *CpLEA5*, the Late Embryogenesis Abundant Protein Gene from *Chimonanthus praecox*, Possesses Low Temperature and Osmotic Resistances in Prokaryote and Eukaryotes. Int. J. Mol. Sci..

