# Supplementary material for: CpLEA5, the Late Embryogenesis Abundant Protein Gene from Chimonanthus praecox, Possesses Low Temperature and Osmotic Resistances in Prokaryote and Eukaryotes
Source: Int J Mol Sci. 2015 Nov 11;16(11):26978–90. doi: 10.3390/ijms161126006 (PMC4661864; doi:10.3390/ijms161126006)
Supplement: Supplementary file 1 [file ijms-16-26006-s001.pdf]

Supplementary Materials: *CpLEA5*, the Late Embryogenesis Abundant Protein Gene from *Chimonanthus praecox*, Possesses Low Temperature and Osmotic Resistances in Prokaryote and Eukaryotes

Yiling liu, Xieli Xia, Xilong Liang and Shihong Zhang

Table S1. Percent identity matrix for strains presented in Figure 1 was performed by alignment program of Clustal Omega [43].

Strain	% Identity									
	Z. mays	*O. sativa*	*C. sinensis*	*C. unshiu*	*T. cacao*	*R. communis*	*C. praecox*	*N. nucifera*	*S. nigrum*	*N. tabacum*
Z. mays	100.00	67.78	35.71	35.71	38.82	38.82	40.51	36.90	45.57	40.74
O. sativa	67.78	100.00	38.10	38.10	45.24	42.35	43.04	38.82	43.59	41.98
C. sinensis	35.71	38.10	100.00	97.94	61.46	61.29	50.00	53.68	48.35	52.13
C. unshiu	35.71	38.10	97.94	100.00	60.42	60.22	47.73	52.63	46.15	50.00
T. cacao	38.82	45.24	61.46	60.42	100.00	64.58	46.74	50.51	47.87	48.96
R. communis	38.82	42.35	61.29	60.22	64.58	100.00	43.18	50.53	42.22	50.54
C. praecox	40.51	43.04	50.00	47.73	46.74	43.18	100.00	53.26	41.18	42.22
N. nucifera	36.90	38.82	53.68	52.63	50.51	50.53	53.26	100.00	51.09	54.74
S. nigrum	45.57	43.59	48.35	46.15	47.87	42.22	41.18	51.09	100.00	67.39
N. tabacum	40.74	41.98	52.13	50.00	48.96	50.54	42.22	54.74	67.39	100.00

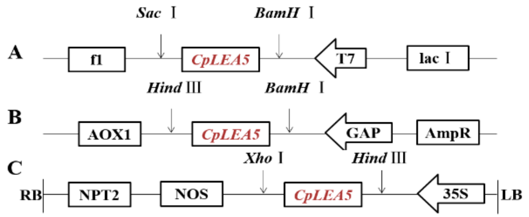

Figure S1. Construction of *CpLEA5*-containing Transformation Vectors. (**A**) The pET-32a::*CpLEA5* vector used for prokaryotic expression; (**B**) The pRUL129::*CpLEA5* vector used for yeast expression; (**C**) T-DNA map of the pTEV7::*CpLEA5* binary vector to transform plants. GAP: GAP promoter; 35S: CaMV 35S promoter; T7: T7 promoter; f1: f1 origin; lac I, lac I coding sequence; NOS, NOS terminator; AmpR, ampicillin resistance gene; NPT2, kanamycin resistance gene.
